# Galectin-1-dependent ceRNA network in HRMECs revealed its association with retinal neovascularization

**DOI:** 10.1186/s12864-023-09352-y

**Published:** 2023-06-15

**Authors:** Ning Yang, Ningzhi Zhang, Zhiyi Wang, Wenye Cao, Xuejun He, Wenxi Zhang, Yiqiao Xing

**Affiliations:** 1 Department of Ophthalmology, Renmin Hospital of Wuhan University, Jiefang Road #238, Wuhan, 430060 Hubei China; 2 Department of Ophthalmology, Aier Eye Hospital of Wuhan University, Wuhan, China

**Keywords:** Competing endogenous RNA, Galectin-1, Long non-coding RNAs, Retinal neovascularization

## Abstract

**Background:**

Retinal neovascularization (RNV) is a leading cause of blindness worldwide. Long non-coding RNA (lncRNA) and competing endogenous RNA (ceRNA) regulatory networks play vital roles in angiogenesis. The RNA-binding protein galectin-1 (Gal-1) participates in pathological RNV in oxygen-induced retinopathy mouse models. However, the molecular associations between Gal-1 and lncRNAs remain unclear. Herein, we aimed to explore the potential mechanism of action of Gal-1 as an RNA-binding protein.

**Results:**

A comprehensive network of Gal-1, ceRNAs, and neovascularization-related genes was constructed based on transcriptome chip data and bioinformatics analysis of human retinal microvascular endothelial cells (HRMECs). We also conducted functional enrichment and pathway enrichment analyses. Fourteen lncRNAs, twenty-nine miRNAs, and eleven differentially expressed angiogenic genes were included in the Gal-1/ceRNA network. Additionally, the expression of six lncRNAs and eleven differentially expressed angiogenic genes were validated by qPCR in HRMECs with or without si*LGALS1*. Several hub genes, such as *NRIR*, *ZFPM2-AS1*, *LINC0121*, *apelin*, *claudin-5*, and *C-X-C motif chemokine ligand 10*, were found to potentially interact with Gal-1 via the ceRNA axis. Furthermore, Gal-1 may be involved in regulating biological processes related to chemotaxis, chemokine-mediated signaling, the immune response, and the inflammatory response.

**Conclusions:**

The Gal-1/ceRNA axis identified in this study may play a vital role in RNV. This study provides a foundation for the continued exploration of therapeutic targets and biomarkers associated with RNV.

**Supplementary Information:**

The online version contains supplementary material available at 10.1186/s12864-023-09352-y.

## Background

Retinal neovascular diseases, such as proliferative diabetic retinopathy, retinopathy of prematurity, and retinal vein occlusion, are the major causes of blindness [1]. Retinal angiogenesis is the process by which new blood vessels sprout from preexisting retinal vessels and invade the retina [2]. During normal retinal vascular development, vascular endothelial cells proliferate and migrate through the extracellular matrix under the action of various cytokines, forming new vessels in a highly ordered manner. The unique vascular supply of the retina as well as the ability to visualize its vasculature in vivo and selectively express its genes make the retina an ideal model system for studying the molecular mechanisms of angiogenesis. Retinal neovascularization (RNV), also known as abnormal angiogenesis, is a key pathological process in ocular blinding diseases [3]. These newly formed blood vessels are fragile and readily lead to retinal edema, leakage, hemorrhage, and detachment, resulting in catastrophic visual impairment and blindness [4]. Angiogenesis is a complex process that depends on precisely regulated gene expression. Several important factors related to angiogenesis have been identified, including vascular endothelial growth factor (VEGF), platelet-derived growth factor, and hypoxia-inducible factors [5–7]. However, the biochemical events responsible for RNV development and progression are not fully understood. Although anti-VEGF therapy is considered a principal clinical treatment regime, the associated therapeutic effects can be insufficient in some patients and are accompanied by a high recurrence rate after a single treatment as well as rapid allergic reactions [8]. Therefore, it is necessary to develop other therapeutic targets and biomarkers. However, to achieve this, studies on the upstream and downstream regulatory mechanisms of these neovascularization-related factors are needed to elucidate additional potential targets for the treatment of ischemic retinopathy.

Recently, an increasing number of studies have found that RNA-binding proteins (RBPs) and non-coding RNAs play important regulatory roles in major physiological and pathological changes [9]. RBPs can regulate basal RNA transcription and post-transcriptional processes, including variable cleavage, modification, transport, translation, degradation, and metabolism by binding to RNA. Abnormal expression, or loss of function, of RBPs leads to the occurrence and development of various diseases [10]. In particular, RBPs have important functions in eye development and ocular diseases, making them potential therapeutic targets for neovascularization [11].

MicroRNAs (miRNAs) are typically ~ 22 nt in length and negatively regulate target messenger RNAs (mRNAs) by promoting mRNA degradation or inhibiting translation. Long non-coding RNAs (lncRNAs) are > 200 nt in length and exhibit no, or limited, protein-coding potential. Accumulating evidence indicates that lncRNAs play crucial regulatory roles in a wide range of biological and pathological processes including angiogenesis [12]. For example, lncRNA-Meg3 knockout mice exhibit increased expression of VEGF pathway genes and increased cortical microvessel density [13]. In fact, many abnormally expressed lncRNAs are associated with eye disorders [14]. For example, *MALAT1* up-regulation is an important pathogenic mechanism of diabetes-induced microvascular dysfunction. Hence, inhibition of *MALAT1* may be a potential target for anti-angiogenic therapy of diabetes-related microvascular complications [15]. Moreover, the lncRNA *TUG1* reduces the expression of VEGFA in OIR mice by competitively adsorbing miR-299, thus participating in the regulation of RNV [16]. Additionally, numerous studies have revealed that competing endogenous RNA (ceRNA) networks participate in gene regulation at multiple levels [17]. lncRNAs are predominantly located in the nucleus, which underscores their regulatory role in gene transcription. Studies have indicated that lncRNAs can be targeted for therapeutic purposes. In particular, the role of ceRNA regulatory networks among lncRNAs, miRNAs, and mRNAs in RNV progression has drawn increasing attention. For instance, knockdown of the lncRNA *MIAT* considerably improves diabetic retinal microvascular dysfunction in vivo and inhibits endothelial cell proliferation, migration, and tube formation in vitro. *MIAT* functions as a competitive endogenous RNA and forms a feedback loop with vascular endothelial growth factor and Mir-150-5p to regulate endothelial cell function [18]. Meanwhile, *MALAT1* knockdown has an important regulatory role in diabetic retinopathy by inhibiting proliferation, migration, tubule formation, and vascular permeability of human retinal microvascular endothelial cells (HRMECs) induced by mercury through up-regulation of Mir-125b [19].

Galectins are expressed in a wide range of species, suggesting their important role in basic cellular mechanisms. Galectin-1 (Gal-1), encoded by *LGALS1*, is a carbohydrate-binding protein that shares a conserved carbohydrate-recognition domain [20]. It has been proposed to mediate cell adhesion and migration and is involved in modulating various aspects of cellular biology, including cell cycle, proliferation, migration, apoptosis, angiogenesis, and cell adhesion [21]. A study indicated that Gal-1 played a central role in maintaining angiogenesis in endometriosis and may be a potential therapeutic target for this disease [22]. We previously demonstrated that si*LGALS1* inhibits pathological RNV and promotes retinal vascular normalization in an oxygen-induced retinopathy mouse model, suggesting its crucial role in triggering vascular signaling programs and mediating the anti-angiogenic treatment response [23]. Recently, several independent experiments showed that Gal-1 could achieve its biological functions, including angiogenesis via binding to lncRNAs and mRNAs, indicating its potential RNA-binding activity [24–26]. In addition, Gal-1 was found to interact with angiogenesis-related mRNA, including *VEGFA*, *EGR1*, and *LAMA5*. Sh*LGALS1* inhibits capillary formation and alters the expression levels of several Gal-1 binding angiogenesis-related mRNAs within in vitro angiogenesis assays [27]. However, the potential downstream mechanisms of Gal-1 in the RNV process have not been extensively studied.

In this study, we explored the potential RNA-binding activity of Gal-1. RBPs are associated with several cellular functions that interact with various RNAs. For instance, RBP FUS can regulate glioma angiogenesis through the FUS/circ_002136/Mir-138-5p/SOX13 feedback loop [28]. Additionally, lncRNA-422 reportedly inhibits the proliferation and growth of colorectal cancer cells by interacting with RBP SFPQ [29]. Meanwhile, cumulative evidence has demonstrated that lncRNAs are involved in nearly all cellular processes. Given the complex function of RBP-lncRNA pairs, aberrantly expressed lncRNAs may be partially responsible for Gal-1-mediated RNV [30]. Continued studies on the function of abnormally expressed RBPs in retinal neovascular diseases and their possible upstream and downstream mechanisms of action are required to develop personalized treatment regimens. Indeed, downstream molecules of Gal-1 may serve as an effective indicator of disease states and might represent a superior therapeutic target for existing protein-coding genes; however, further in-depth research is required to examine the toxicity and pharmacokinetics of lncRNAs.

Herein, we construct a comprehensive regulatory network of lncRNAs, miRNAs, and mRNAs in siLGALS-knockdown HRMECs. This study describes a previously unrecognized relationship between Gal-1 and the ceRNA regulatory network and provides a foundation for the development of a novel treatment for neovascularization-related diseases.

## Results

### LGALS1 regulates the expression of genes associated with angiogenesis in HRMECs

**Fig. 1:**
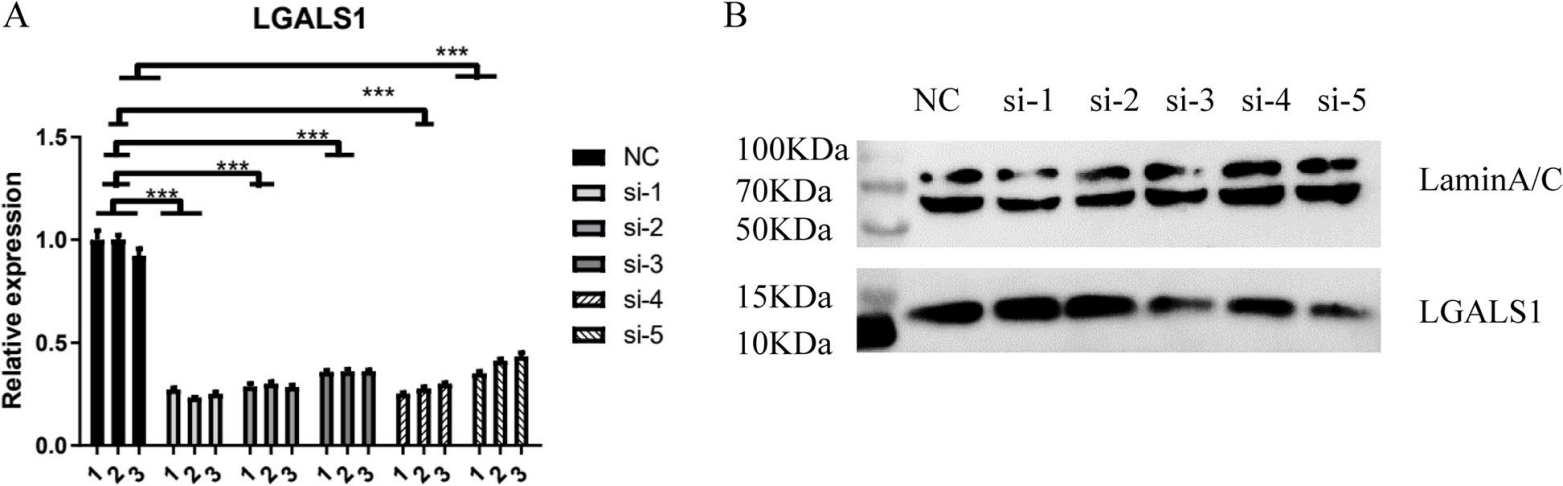
The effects of si*LGALS1* in HRMECs. **a** *LGALS1* expression in normal control and si*LGALS1* groups quantified by qPCR. **b** *LGALS1* expression in normal control and si*LGALS1* groups quantified by Western blot. ***p < 0.001

**Fig. 2:**
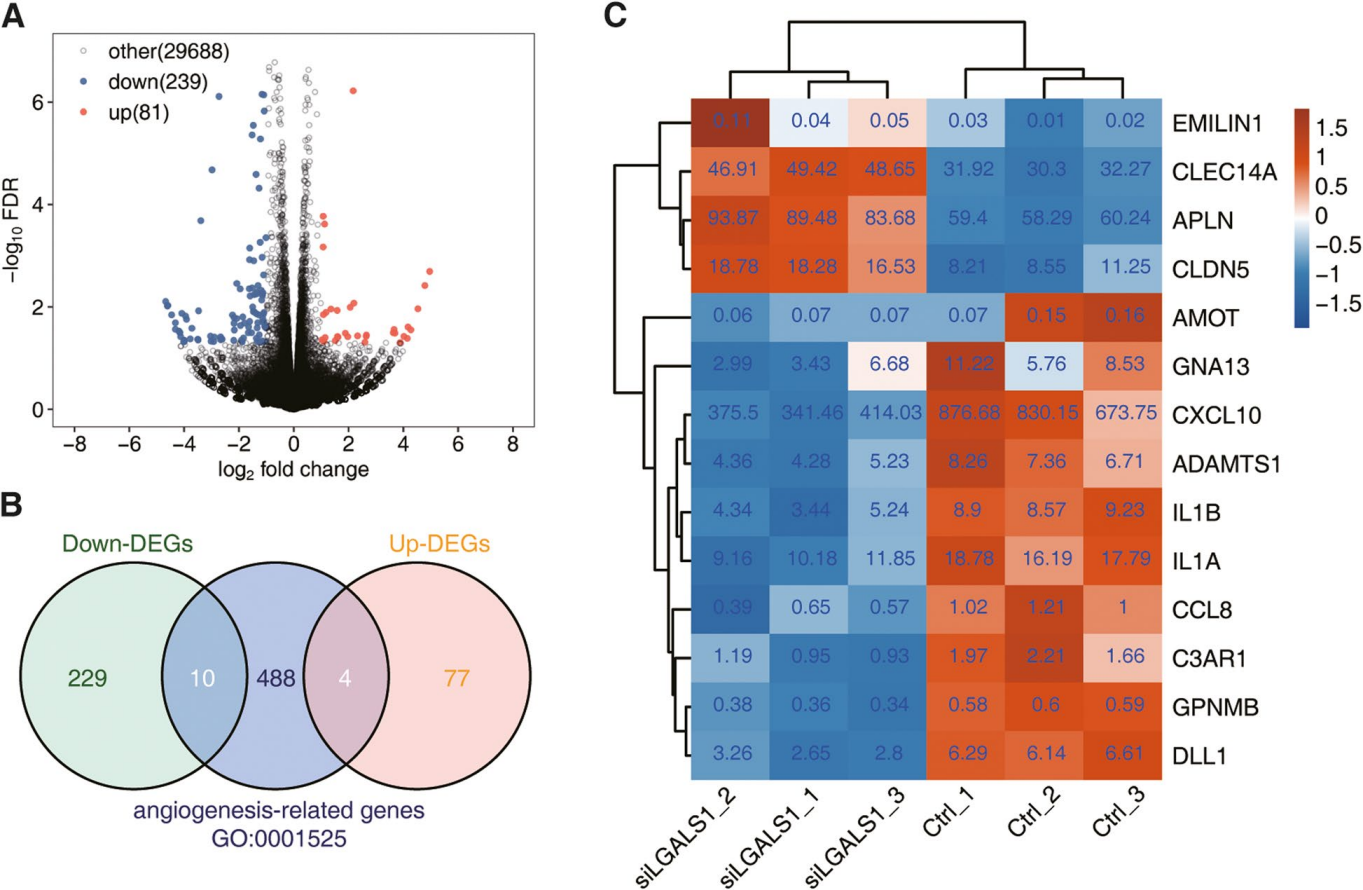
*LGALS1* regulates the expression of genes associated with angiogenesis in HRMECs. **a** Differences between the si*LGALS1* and control groups (fold change; FC ≥ 1.5 or ≤ 2/3, *p*-value ≤ 0.05). **b** Overlap between DEGs and angiogenesis-related genes. A total of 502 angiogenesis-related genes were extracted from the GO Ontology database (GO:0,001,525). **c** Expression profiles of differentially expressed (DE) angiogenesis-related genes. FPKM of each gene was labeled in the heatmap

We successfully constructed si*LGALS1* HRMECs models for further investigation (Fig. 1). Si-1, si-2, si-3, si-4, and si-5 groups showed considerable down-regulation in *LGALS1* mRNA, and si-3, si-4, and si-5 groups showed considerable down-regulation in Gal-1 protein levels. The original, unprocessed electrophoretic gel images are shown in the Supplementary file (Fig. S1). Taking all the results into account, we finally chose si-4 group to continue the experiments. We analyzed differentially-expressed genes (DEGs) between the *LGALS1*-silenced group (si*LGALS1*) and the control group (Ctrl) in HRMECs (Fig. 2a). Compared with those in the Ctrl, the downregulated genes in the si*LGALS1* group were nearly thrice the number of upregulated genes (Fig. 2b). Upregulated genes were primarily concentrated in G protein-coupled receptor signaling pathway, cellular adhesion, and transcriptional regulation by RNA polymerase II pathway according to GO analysis, whereas downregulated genes were mainly concentrated in inflammatory response and chemokine-mediated signaling pathway (Fig. S2).

Here, we focused on DEGs associated with angiogenesis. Through DEGs and gene overlap contained in the gene ontology (GO) term of angiogenesis (GO:0,001,525), we found a considerable change in 14 angiogenesis-related genes, among which 10 were upregulated and 4 were downregulated (Fig. 2c). The 14 angiogenesis-related genes included *EMILIN1*, *CLEC14A*, *APLN*, and *CLDN5* (downregulated) and *AMOT*, *GNA13*, *C-X-C motif chemokine ligand 10* (*CXCL10*), *ADAMTS1*, *IL1B*, *IL1A*, *CCL8*, *C3AR1*, *GPNMB*, and *DLL1* (upregulated).

### Analysis of differentially expressed lncRNAs between *LGALS1*-knockdown and control HRMECs

*LGALS1* may influence angiogenesis by regulating lncRNA expression [27, 31]. However, the Gal-1-lncRNA interaction map in angiogenesis remains unclear. *LGALS1*-associated lncRNAs were identified and analyzed in the current study.

**Fig. 3:**
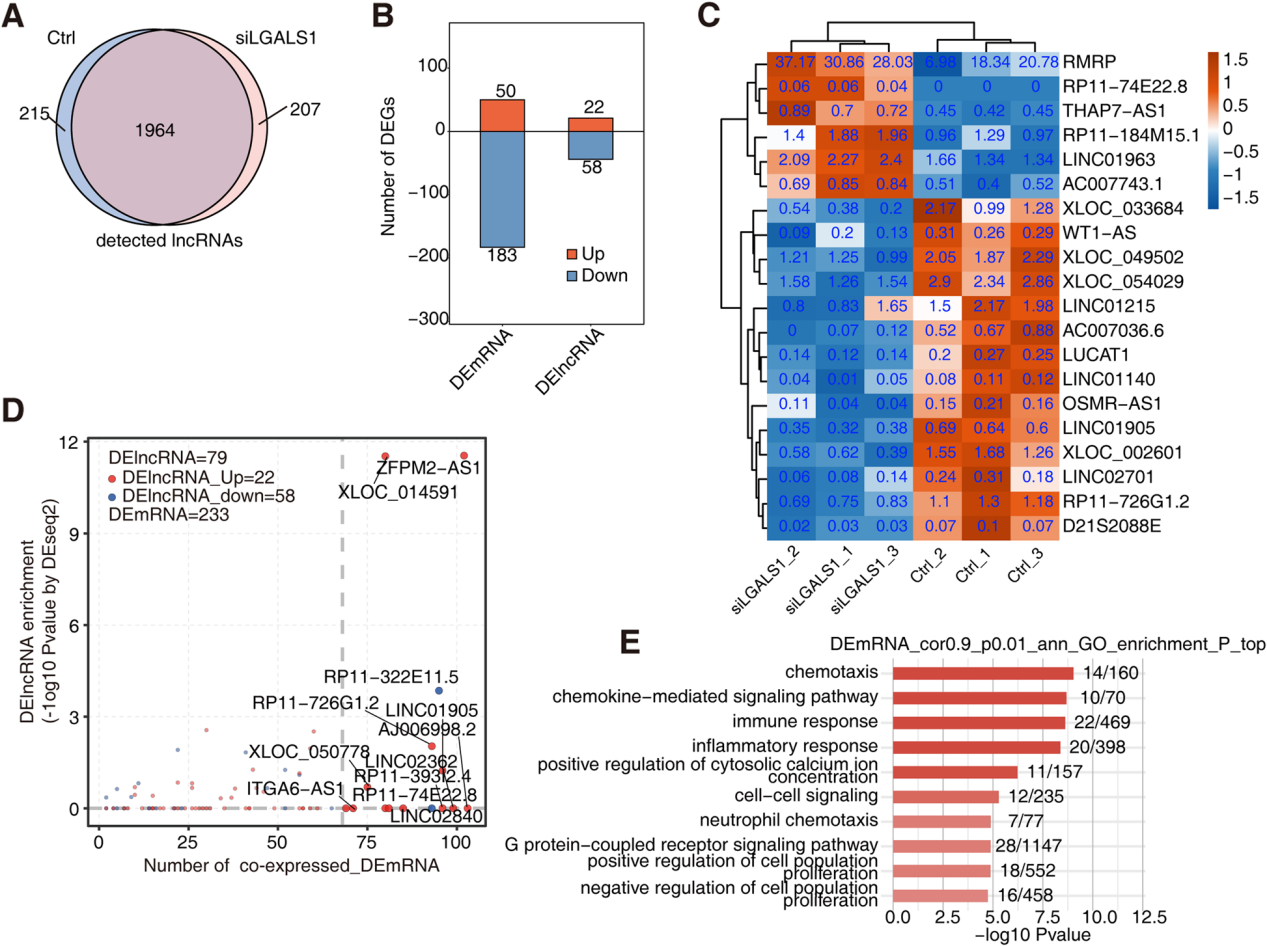
Analysis of differentially expressed lncRNAs between *LGALS1* knockdown and control samples in HRMECs. **a** Number of detected lncRNAs. The known and new predicted lncRNAs were combined. LncRNAs with FPKM ≥ 0.2 in at least one sample were considered to be detected in this group. **b** Number of up-regulated and down-regulated annotated mRNAs and lncRNA (DElncRNA). DESeq2 was used for differential expression analysis with criteria of *p-*value ≤ 0.05 and fold change (FC) ≥ 1.5 or ≤ 2/3. **c** Expression profile of the top 20 most significant DE lncRNAs ordered by *p*-value with their corresponding FPKM. **d** DE lncRNAs and the number of their co-expressed DE mRNAs. Red points denote up-regulated lncRNAs involved in co-expression pairs and blue points denote down-regulated lncRNAs. *p*-value cutoffs ≤ 0.01 and Pearson coefficient of ≥ 0.9 were applied to identify the co-expression pairs. **e** Top 10 most enriched GO terms (biological process) by DE mRNAs co-expressed with the DE lncRNAs between si*LGALS1* and control samples

The known and newly predicted lncRNAs were combined and a Venn diagram of the detected known lncRNAs and novel lncRNAs in si*LGALS1* was constructed (Fig. 3a). LncRNAs with fragments per kilobase of exon per million (FPKM) ≥ 0.2 in at least one sample were considered to be detected in this group (Fig. S3). In total, 65 lncRNAs were significantly altered in HRMECs with *LGALS1* knockdown compared with those in the control HRMECs (fold change [FC] ≥ 1.5 or ≤ 2/3, P ≤ 0.05). Among these, 21 upregulated and 44 downregulated annotated lncRNAs were identified through differential analysis in si*LGALS1* cells (Fig. 3b). The heatmap shows the expression profiles of the top 20 most significant differentially expressed (DE) lncRNAs (Fig. 3c). Based on the number of co-expressed DE lncRNAs and DE mRNAs between si*LGALS1* and control samples (Fig. 3d), the top 10 most enriched GO terms (biological processes) revealed that DE mRNAs may be closely associated with chemotaxis, immune responses, and inflammatory responses (Fig. 3e). The co-expression network between DE lncRNAs and DE mRNAs, which are involved in the 10 GO terms shown in red (Fig. S3g), was constructed. *LINC01905*, *AJ006998.2*, *RP11-39,312.4*, *LINC02362*, *ZFPM2-AS1*, *RP11-726G1.2*, *RP11-322E11.5*, *D21S2088E*, *XLOC_OO2601*, and *RP11-74E22.8* were included. The top 10 most enriched Kyoto Encyclopedia of Genes and Genomes (KEGG) pathways by DE mRNAs co-expressed with DE lncRNAs between si*LGALS1* and the control samples are demonstrated (Fig. S3f). The top three pathways were cytokine-cytokine receptor interaction, viral protein interaction with cytokine and cytokine receptor, and toll-like receptor signaling pathway.

### Regulatory network of lncRNA-miRNA-angiogenesis genes mediated by *LGALS1* deregulation in HRMEC

**Fig. 4:**
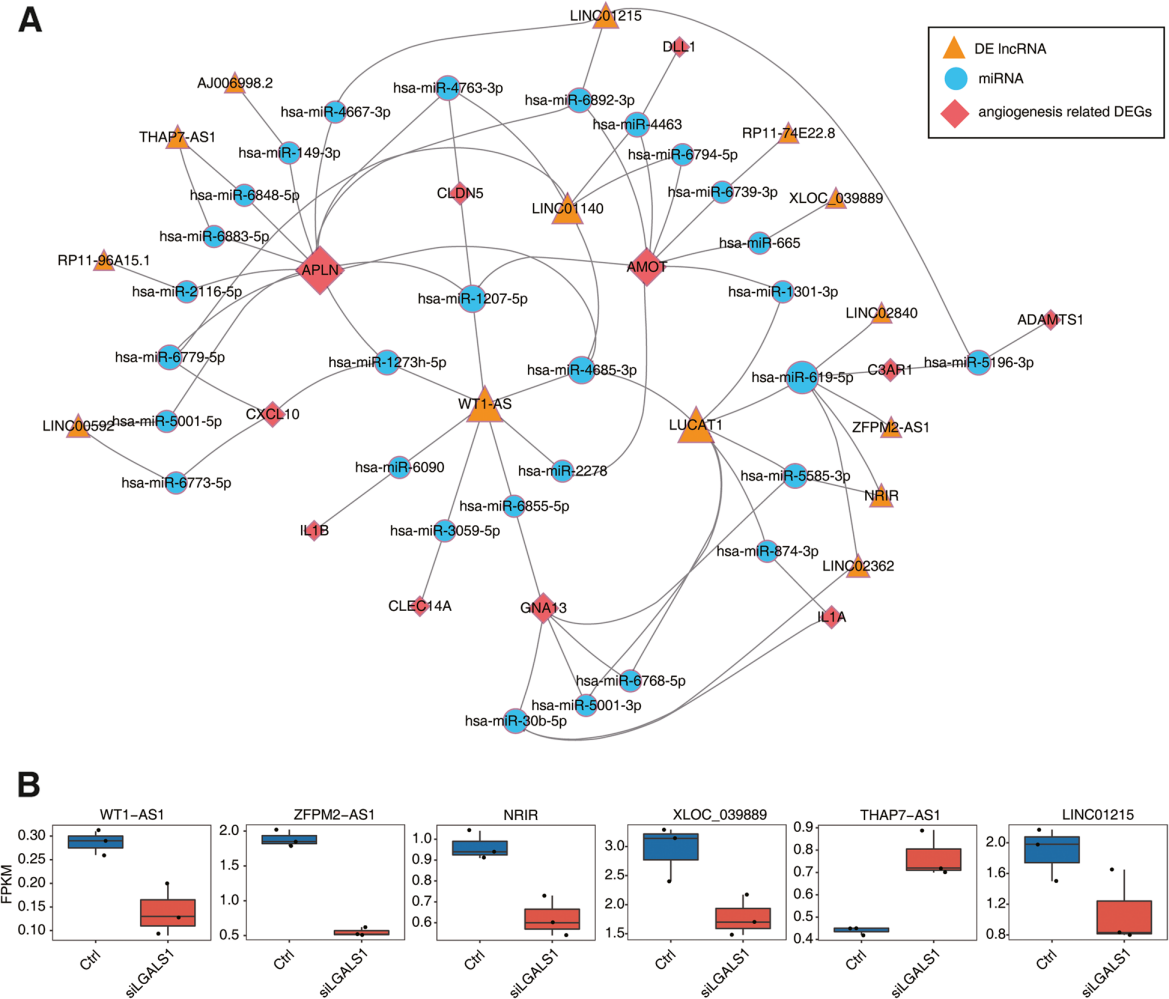
Regulatory network of lncRNA-miRNA-angiogenesis genes mediated by *LGALS1* deregulated in HRMEC. **a** Regulatory network of lncRNA-miRNA-angiogenesis genes. The orange triangles represent DE lncRNAs, blue circles represent miRNAs, red diamonds represent angiogenesis-related differential genes, and gray line represents the target relationship between miRNA and lncRNA or mRNA. The larger the node, the more other nodes are associated. **b** Expression profile of six DE lncRNAs involved in the network

We constructed co-expression network of DE lncRNAs and DE angiogenesis-related genes (Fig. S4a). Eight DE lncRNAs (*LINC02840*, *AJ006998.2*, *RP11-74E22.8*, *RP11-96A15.1*, *LUCAT1*, *LINC02362*, *LINC00592*, and *LINC01140*) involved in the regulatory network were further verified (Fig. S4b). LncRNAs act as sponges of related miRNAs. Together, lncRNAs and miRNAs form an lncRNA–miRNA–angiogenesis-related gene axis mediated by *LGALS1* deregulation in HRMECs. A regulatory network was established, comprising 14 lncRNAs, 29 miRNAs, and 11 angiogenic DEGs, and a network diagram was drawn using Cytoscape (Fig. 4a). The lncRNA-miRNA-mRNA pathways in this regulatory network diagram were selected for validation and follow-up studies. Expression profile supported six lncRNAs (*WT1-AS1*, *ZFPM2-AS1*, *NRIR*, *XLOC_039889*, *THAP7-AS1*, and *LINC01215*) as DE lncRNAs (Fig. 4b). Some DE lncRNAs were situated in the center of co-expression networks which more likely have an impact on angiogenesis regulation in si*LGALS1* HRMECs. Among these angiogenesis-related genes, expression of *APLN*, *CLDN5*, and *CLEC14A* were up-regulated after si*LGALS1* and they may participate in G protein-coupled receptor signaling pathway, cell adhesion, and neuroactive ligand-receptor interaction (Fig. S2a, c). Meanwhile, expression of *AMOT*, *GNA13*, *IL1B*, and *CXCL10* were down-regulated in si*LGALS1* cells and they may impact inflammatory response, chemokine-mediated signaling pathway, and viral protein interaction with cytokine and cytokine receptor (Fig. S2b, d).

### Validation of the expression of lncRNA and angiogenesis-related genes in HRMECs with or without knockdown *LGALS1*

**Fig. 5:**
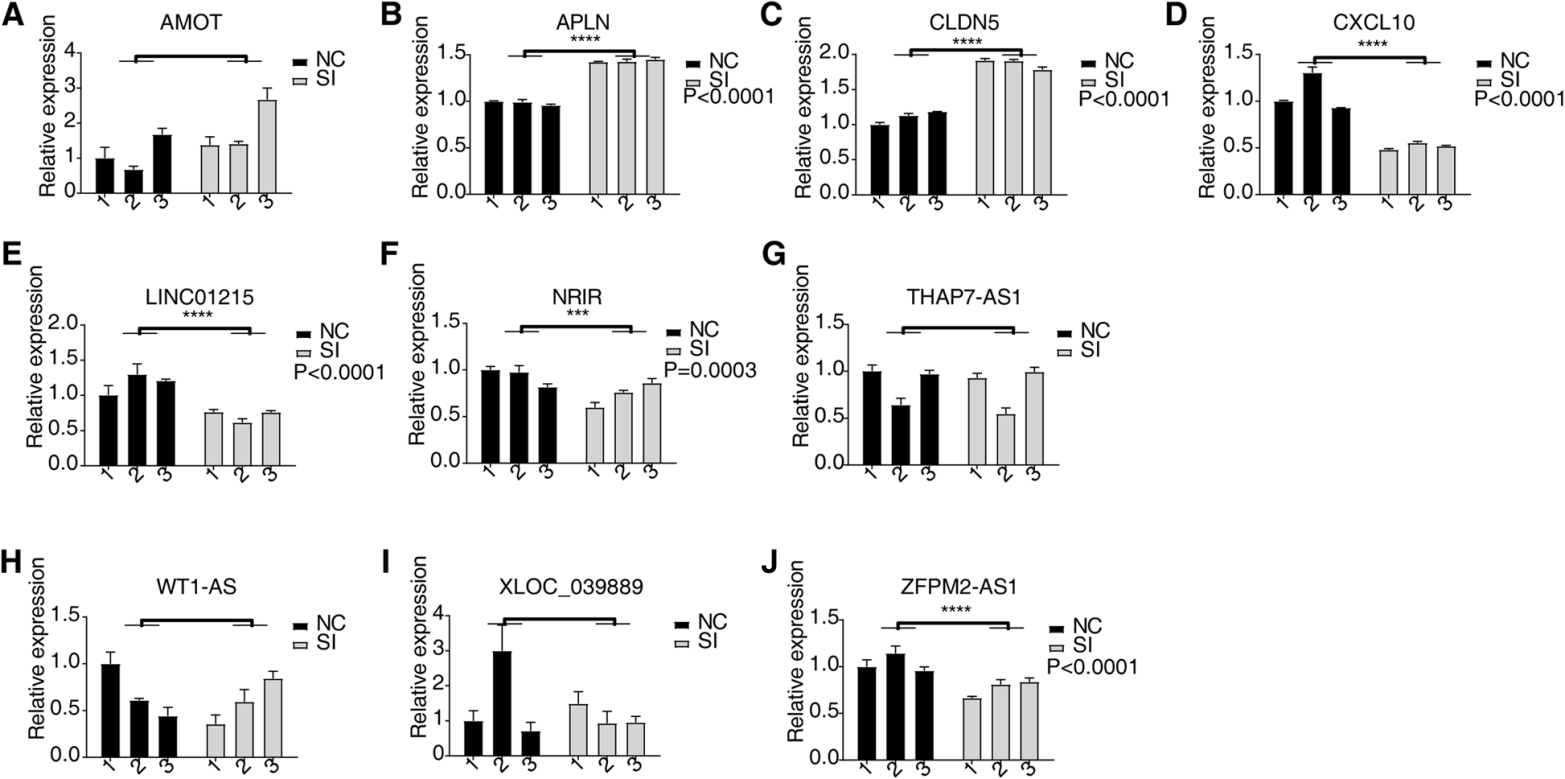
Validation of the expression of lncRNA and angiogenesis-related genes in HRMECs with or without knockdown *LGALS1*. **a-d** Expression of differentially expressed angiogenesis-related genes involved in the network quantified by qPCR. **e-j** Expression of differentially expressed lncRNAs involved in the network quantified by qPCR. ***p < 0.001, ****p < 0.0001

We further verified 6 crucial lncRNAs (down-regulated expression: *NRIR*, *ZFPM2-AS1*, and *LINC0121*; no significant differences: *WT1-AS*, *THAP7-AS1*, *XLOC_039889*) and 11 angiogenesis-related mRNAs (down-regulated expression: *IL1A*, *GNA13*, *IL1B*, *ADAMTS1*, *CXCL10*, *C3AR1*, *DLL1*; up-regulated expression: *APLN*, *CLEC14A*, *CLDN5*; no significant differences: *AMOT*) by qPCR in HRMECs with or without knockdown *LGALS1* (Fig. 5 and Fig. S5).

## Discussion

Gal-1 is an endogenous lectin that exists in peripheral lymphoid organs and inflammatory sites. Several studies have demonstrated an intricate interplay between Gal-1 and lncRNAs. To illustrate, the lncRNA *ESCCAL*-1 may promote cell cycle progression by interacting with, and stabilizing, Gal-1 in esophageal squamous cell carcinoma [25]. Moreover, the lncRNA small nucleolar RNA host gene 22 (*SNHG22*) impacts cancer initiation and progression via the miR-2467/Gal-1 signaling pathway [32]. Nevertheless, the mechanism of action of most ncRNAs, as well as their interaction with RBPs, remain to be elucidated; in particular, the role of Gal-1-ncRNA interaction in the regulation of retinal neovascularization remains to be explored.

In the current study, we systemically profiled lncRNA expression and analyzed DE lncRNAs and neovascularization-related genes between *LGALS1* knockdown and control groups in HEMECs using next-generation sequencing. We found a marked change in 14 angiogenesis-related genes, among which 10 were upregulated and 4 were downregulated (Fig. 2c). *Apelin (APLN), angiomotin (AMOT), CXCL10,* and *claudin 5 (CLDN5)* were located in the central nodes of the network (Fig. 4a). *APLN* is a recently discovered regulator of neovascularization that binds to the APJ receptor to promote proliferation, differentiation, and migration of vascular endothelial cells, while also forming lumen-like structures. *APLN* also plays an important role in retinal vascularization [33], while also impacting the progression of osteoarthritis by regulating VEGF-dependent angiogenesis and mir-150-5p expression [34]. Meanwhile, deletion of *AMOT* can inhibit the migration and expansion of physiological and pathological vascular networks [35]. In addition, the lncRNA *UCA1* may directly regulate *AMOT* to activate target genes in epithelial ovarian cancer, thus highlighting a potential interaction between *AMOT* and lncRNAs [36]. Additionally, *CXCL10* plays a critical role in vascular remodeling following spinal cord injury, whereas angiogenesis is enhanced following anti-*CXCL10* treatment of spinal cord injuries [37]. *CLDN5*, a tight junction protein, is vital for maintaining integrity of endothelial cells and vasostabilizing [38]. It also participates in regulating permeability of the blood-tumor barrier via lncRNA *NEAT1*/miR-181d-5p-mediated expression changes [39]. We picked several genes to conduct further vertification. Interestingly, the expression levels of *APLN* and *CLDN5* were elevated, while the expression level of *CXCL10* was reduced. However, the expression level of *AMOT* showed no significant tendency. This possibly indicates that increased sample numbers are warranted to eliminate result deviation that may occur due to insufficient sample size.

The CeRNA network plays an important role in the regulation of retinal angiogenesis [40]. In fact, the ceRNA hypothesis suggests a new mechanism of interaction among RNAs, in which lncRNAs block the binding of miRNAs to mRNAs by competitively sponging miRNAs, thereby regulating mRNA expression. Understanding this system of RNA communication will provide new insights into gene regulatory networks and mechanisms of human disease [41]. We examined lncRNA-miRNA interactions to reveal the possible downstream regulatory mechanism of Gal-1 and constructed an lncRNA–angiogenesis-related mRNA co-expression network. Our results show that Gal-1 may be involved in regulating biological processes related to chemotaxis, chemokine-mediated signaling pathway, immune response, inflammatory response, positive regulation of cytosolic calcium ion concentration, cell–cell signaling, neutrophil chemotaxis, G protein-coupled receptor signaling pathway, and regulation of cell population proliferation (Fig. 3e). This is consistent with the reported function of Gal-1, that is, participating in inflammatory regulation and affecting both cell proliferation as well as chemotaxis [42, 43]. These pathways are closely related to the formation of new blood vessels. Gal-1 has emerged as a multifaceted protein that exerts a wide spectrum of regulatory effects both intracellularly and extracellularly [44]. Whether Gal-1 can regulate RNV by acting either extracellularly through specific binding to cell surface glycan structures or intracellularly through modulation of pathways remains largely unexplored and is worth further exploration. Moreover, whether some agents, such as lactose, that inhibit the binding of galectins to the cell surface, or recombinant Gal-1 would affect the expression of these differentially expressed genes, when added to the cells, is worth further exploration. In the present study, we hypothesized that Gal-1 could bind RNA directly and focused on its potential intracellular regulatory functions first.

We also predicted key lncRNAs and miRNAs that may regulate the expression of angiogenesis-related genes. These key lncRNAs and miRNAs were then screened as potential prognostic biomarkers and therapeutic targets for angiogenesis-related diseases. We further verified the most likely crucial lncRNAs (*WT1-AS*, *NRIR*, *ZFPM2-AS1*, *LINC01215*, *THAP7-AS1*, and *XLOC_039889*). *NRIR*, *ZFPM2-AS1*, and *LINC01215* expression levels were down-regulated in si*LGALS1* HEMECs. *ZFPM2-AS1* has been shown to play crucial roles in the progression of retinoblastoma, an angiogenesis-associated disease [45]. The interferon (IFN) family of cytokines serves as pivotal regulators of angiogenesis [46], while NRIR was found to be a negative Regulator of IFN Response [47, 48]. Tumor growth of Clear Cell Renal Cell Carcinoma was suppressed by silenced *LINC01215* via miR-184 [49]. However, whether it can regulate RNV by ceRNA network remains unknown. Our study revealed lncRNA-miRNA-mRNA co-expression regulatory networks in si*LGALS1* HRMECs, indicating the possible downstream modulation mechanism of Gal-1 in RNV. Whether the regulatory role of the ceRNA network itself is really valid when Gal-1 is normally expressed needs further exploration. Collectively, these findings suggest that the Gal-1/ceRNA pathway is a promising target for the diagnosis and treatment of neovascularization-associated diseases. More specifically, regulating the expression of both Gal-1 and downstream lncRNAs may maximize the efficiency of neovascularization inhibition.

## Conclusions

In conclusion, our research provides insights regarding the possible Gal-1/ceRNA regulatory network in HRMECs with si*LGALS1*. However, additional studies elucidating the ceRNA regulatory networks requires validation. This lncRNA-miRNA-mRNA network may provide clues to the regulatory pathways in the RNV for the cascade-amplifying synergistic effects of Gal-1. Verification of this hypothesis is expected to further improve the understanding of the molecular mechanism underlying retinal neovascularization at the gene and molecular levels, thereby providing a new target for the prevention and treatment of retinal neovascularization diseases and providing a theoretical basis for the clinical inhibition of neovascularization and promotion of ischemic retinal reperfusion.

## Methods

### Cell culture and transfection

HRMECs lines (Angio-Proteomie, Boston, Mass, USA) were cultured at 37 °C with 5% CO_2_ in ECM supplemented with 10% fetal bovine serum (FBS), 100 µg/mL streptomycin, and 100 U/mL penicillin. To silence the expression of *LGALS1* in HRMECs, an siRNA-containing plasmid was constructed using a vector. Transfected cells were harvested after 48 h for RT-qPCR. All siRNA duplexes were purchased from Gemma (Suzhou, China). Non-targeting control siRNA (siNegative): 5′-UUCUCCGAACGUGUCACGUTT-3′ (sense). siRNA targeting *LGALS1* (si*LGALS1*): CCAGCAACCUGAAUCUCAATT (sense). siRNA transfection of HRMECs was performed using Lipofectamine RNAiMAX Transfection Reagent (Invitrogen, Carlsbad, CA, USA), according to the manufacturer’s protocol.

### Assessment of gene expression

#### qPCR

*GAPDH* (glyceraldehyde-3-phosphate dehydrogenase) was used as a control gene to assess the effects of *LGALS1* knockdown. cDNA synthesis was performed using standard procedures, and RT-qPCR was performed on a Bio-Rad S1000 with Hieff™ qPCR SYBR® Green Master Mix (Low Rox Plus; YEASEN, Shanghai, China). Information on the primers is presented in Table S1. The concentration of each transcript was normalized to the *GAPDH* mRNA level using the 2^−ΔΔCT^ method [50].

#### Western blotting

HRMECs were lysed in ice-cold wash buffer (1 × PBS, 0.1% SDS, 0.5% NP-40, and 0.5% sodium deoxycholate) supplemented with protease inhibitor cocktail (Roche) and incubated on ice for 30 min. Samples were boiled for 10 min in boiling water with 1X SDS sample buffer and separated using 10% SDS-PAGE. Membranes were incubated with TBST buffer (20 mM Tris-buffered saline and 0.1% Tween-20) containing 5% non-fat milk powder for 1 h at room temperature. Membranes were incubated with primary antibody: LGALS1 antibody (A18040, 1:1,000, ABclonal, Boston, MA, USA) and actin (10,298–1-AP, 1:2000, ABclonal, Boston, MA, USA), followed by incubation with HRP-conjugated secondary antibody. Bound secondary antibodies (anti-rabbit 1:10,000, Abcam, Cambridge, England) were detected using an enhanced chemiluminescence (ECL) reagent (Bio-Rad, CA, USA).

### RNA isolation

Total RNA was extracted using TRIZOL (Ambion, Austin, Texas, USA) and purified using two phenol–chloroform treatments followed by treated with RQ1 DNase (Promega, Madison, WI, USA) to remove DNA. The quality and quantity of the purified RNA were determined by measuring the absorbance at 260 nm/280 nm (A260/A280) using a Nanodrop spectrophotometer (Thermo, Waltham, MA, USA). RNA integrity was verified by 1.0% agarose gel electrophoresis.

### Reads alignment and DEG analysis

Clean reads were aligned to the human GRch38 genome using HISAT2 [51]. Uniquely mapped reads were used to calculate the read number and FPKM mapped for each gene. The expression levels of these genes were evaluated by FPKM. DEseq2 models were employed to detect DEGs. Briefly, the original reads were used and a scale factor was applied to explain the difference in library depth. DEseq2 then estimated gene dispersion and reduced the estimates to generate more accurate dispersion estimates to model the read count. Finally, the model of the negative binomial distribution was fitted using DEseq2, and the hypothesis was tested using the Wald test or likelihood ratio test. DEseq2 can be used to analyze the differential expression between two or more samples, and the analysis results can be used to determine whether a gene is differentially expressed by FC, absolute ratio of expression change, and *p*-value. The criteria for significant differential expression were: FC ≥ 1.5 or ≤ 0.66, *p*-value ≤ 0.05.

### LncRNA prediction and direction identification

To systematically analyze the lncRNA expression pattern, we used a pipeline for lncRNA identification similar to that previously reported [52], which was constructed based on the StringTie software [53]. The predicted lncRNAs were compared to the noncoding RNA database (NONCODEv6, http://www.noncode.org/) to identify the lncRNAs that were already present.

### Construction of regulatory lncRNA-miRNA-angiogenesis-related gene networks

First, co-expression analysis was performed for 80 differentially expressed lncRNAs and 14 differentially expressed angiogenesis-related genes, requiring an absolute value of Pearson correlation coefficient ≥ 0.95 and *p* value ≤ 0.01. LncRNAs and angiogenesis-related genes were identified in this study. Then, according to the TargetScan (http://www.targetscan.org/vert_80/) and miRDB (http://mirdb.org/) databases, miRNAs that can target the angiogenesis genes were obtained. Transcription sequences of lncRNAs were extracted, and miRanda (score ≥ 150) and RNAhybrid (*p*-value ≤ 0.05) were used to predict the target of miRNAs. Finally, the results of the two methods were compared, and partial miRNAs and targeted lncRNAs were identified. A regulatory network was established for lncRNAs, miRNAs, and angiogenesis-related genes using Cytoscape (https://cytoscape.org/).

### Gene Ontology and KEGG pathway enrichment analyses

To sort assess functional categories of DEGs, GO terms and KEGG pathways were identified using KOBAS 2.0 [54]. The hypergeometric test and Benjamini–Hochberg FDR control procedure were used to define the enrichment of each term.

### Other statistical analysis

The pheatmap package (https://cran.r-project.org/web/packages/pheatmap/index.html) in R was used to perform clustering based on Euclidean distance. The Student’s *t*-test was used for comparison between the two groups.

## Supplementary Information

**Additional file 1:** **Fig S1.** LGALS1 expression in normal control and siLGALS1 groups quantified by Western blot. a, b The original, unprocessed electrophoretic gel images. **Fig S2.** LGALS1 regulates the expression of genes associated with angiogenesis in HRMECs. a Top 10 most enriched Gene Ontology (GO) terms associated with upregulated genes between the siLGALS1 and control groups. b Top 10 most enriched GO terms associated with downregulated genes between the siLGALS1 and control groups. c Top 10 most enriched Kyoto Encyclopedia of Genes and Genomes (KEGG) terms associated with upregulated genes between the siLGALS1 and control groups. d Top 10 most enriched KEGG terms of downregulated genes between the siLGALS1 and control groups. HRMECs, human retinal microvascular endothelial cells. **Fig S3.** Analysis of differentially expressed lncRNAs in HRMECs between LGALS1-knockdown and control groups. a Detected known lncRNAs (left panel) and novel lncRNAs(right panel) in the siLGALS1 and control groups. LncRNAs with FPKM ≥ 0.2 in at least one sample were considered differentially expressed. b Distribution of exon counts of known lncRNAs, novel lncRNAs, and protein-coding RNAs. c Distribution of exon lengths of known lncRNAs, novel lncRNAs, and protein-coding RNAs. d Density of the length distribution of known lncRNAs, novel lncRNAs, and protein-coding RNA. The length density distribution was generated using the density function in R software. e Expression profile of DE lncRNAs. f Top 10 most enriched KEGG pathways associated with DE mRNAs co-expressed with DE lncRNAs in the siLGALS1 and control groups. g Co-expression network of DE lncRNAs and DE mRNAs associated with the top 10 GO terms, shown in red in f. LncRNAs are on the left, co-expressed mRNAs are in the center, and enriched GO terms associated with mRNAs are on the right. DE, differentially expressed; HRMECs, human retinal microvascular endothelial cells; FPKM, fragments per kilobase of exon per million fragments mapped; KEGG, Kyoto Encyclopedia of Genes and Genomes; GO, Gene Ontology. **Fig S4.** Regulatory network of lncRNA–miRNA–angiogenesis-related genes mediated by LGALS1 deregulation in HRMECs. a Co-expression network of DE lncRNAs and DE angiogenesis-related genes. Orange triangles indicate lncRNAs, and red rhombuses indicate angiogenesis-related genes. Cutoffs of *p* ≤ 0.01 and PCC ≥ 0.95 were applied to identify co-expression pairs. b Box plots showing the expression profiles of the eight DE lncRNAs involved in the regulatory network. HRMECs, human retinal microvascular endothelial cells; DE, differentially expressed; PCC, Pearson’s correlation coefficient. **Fig S5.** Validation of the expression of angiogenesis-related genes in HRMECs with or without knockdown LGALS1. a-g Expression of differentially expressed angiogenesis-related genes involved in the network quantified by qPCR. ****p < 0.0001. **Table S1.** Primer sequences. **Table S2.** siRNA sequences.

## Data Availability

The datasets generated and analyzed during the current study are available in the Gene Expression Omnibus (GEO, https://www.ncbi.nlm.nih.gov/geo/) database (Accession Number: GSE221037) (The following secure token has been created to allow review of record GSE221037: kxwfoiqcbngvnaf); TargetScan (http://www.targetscan.org/vert_80/); miRDB (http://mirdb.org/); Gene Ontology(http://geneontology.org/); Kyoto Encyclopedia of Genes and Genomes (http://www.kegg.jp/); NONCODE (http://www.noncode.org/).

## Abbreviations

AMOT: Angiomotin
APLN: Apelin
ceRNA: Competing endogenous RNA
CLDN5: Claudin 5
CXCL10: C-X-C motif chemokine ligand 10
DE: Differentially expressed
DEG: Differentially expressed gene
FBS: Fetal bovine serum
FC: Fold change
FPKM: Fragments per kilobase of exon per million
Gal-1: Galectin-1
GO: Gene ontology
KEGG: Kyoto Encyclopedia of Genes and Genomes
lncRNA: Long non-coding RNA
miRNA: MicroRNA
mRNA: Messenger RNA
PCC: Pearson’s correlation coefficient
RBPs: RNA-binding proteins
RNV: Retinal neovascularization
SNHG22: Small nucleolar RNA host gene 22
VEGF: Vascular endothelial growth factor
