# Supplementary material for: Galectin-1-dependent ceRNA network in HRMECs revealed its association with retinal neovascularization
Source: BMC Genomics. 2023 Jun 15;24:327. doi: 10.1186/s12864-023-09352-y (PMC10268405; doi:10.1186/s12864-023-09352-y)
Supplement: Supplementary file 1 — Additional file 1: Fig S1. LGALS1 expression in normal control and siLGALS1 groups quantified by Western blot. a, b The original, unprocessed electrophoretic gel images. Fig S2. LGALS1 regulates the expression of genes associated with angiogenesis in HRMECs. a Top 10 most enriched Gene Ontology (GO) terms associated with upregulated genes between the siLGALS1 and control groups. b Top 10 most enriched GO terms associated with downregulated genes between the siLGALS1 and control groups. c Top 10 most enriched Kyoto Encyclopedia of Genes and Genomes (KEGG) terms associated with upregulated genes between the siLGALS1 and control groups. d Top 10 most enriched KEGG terms of downregulated genes between the siLGALS1 and control groups. HRMECs, human retinal microvascular endothelial cells. Fig S3. Analysis of differentially expressed lncRNAs in HRMECs between LGALS1-knockdown and control groups. a Detected known lncRNAs (left panel) and novel lncRNAs(right panel) in the siLGALS1 and control groups. LncRNAs with FPKM ≥ 0.2 in at least one sample were considered differentially expressed. b Distribution of exon counts of known lncRNAs, novel lncRNAs, and protein-coding RNAs. c Distribution of exon lengths of known lncRNAs, novel lncRNAs, and protein-coding RNAs. d Density of the length distribution of known lncRNAs, novel lncRNAs, and protein-coding RNA. The length density distribution was generated using the density function in R software. e Expression profile of DE lncRNAs. f Top 10 most enriched KEGG pathways associated with DE mRNAs co-expressed with DE lncRNAs in the siLGALS1 and control groups. g Co-expression network of DE lncRNAs and DE mRNAs associated with the top 10 GO terms, shown in red in f. LncRNAs are on the left, co-expressed mRNAs are in the center, and enriched GO terms associated with mRNAs are on the right. DE, differentially expressed; HRMECs, human retinal microvascular endothelial cells; FPKM, fragments per kilobase of exon per million fragments mappe [file 12864_2023_9352_MOESM1_ESM.docx]

**Additional Files**


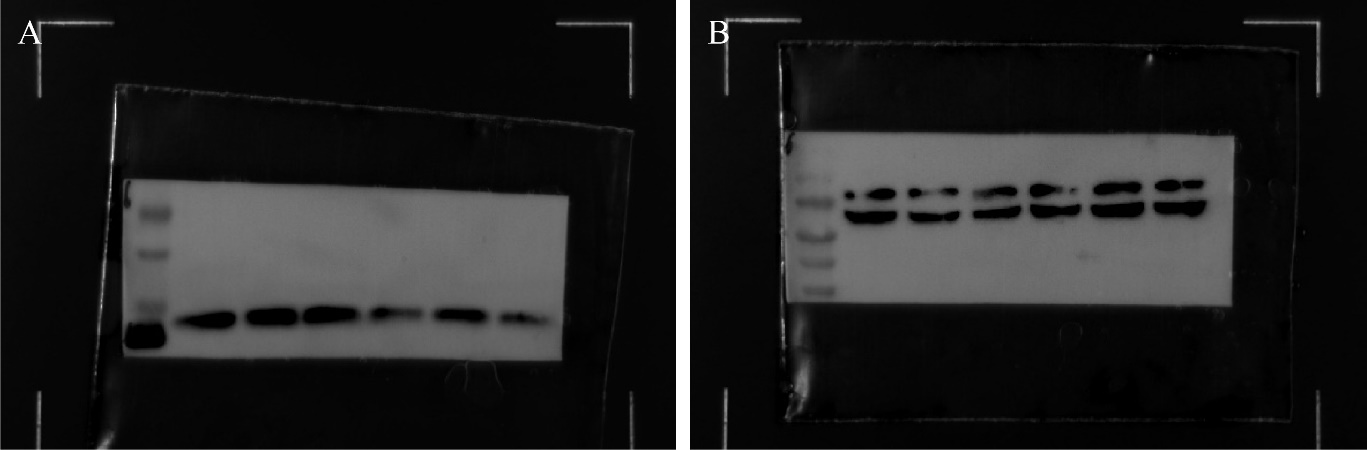


**Fig S1. LGALS1 expression in normal control and siLGALS1 groups quantified by Western blot. a, b** The original, unprocessed electrophoretic gel images.

**
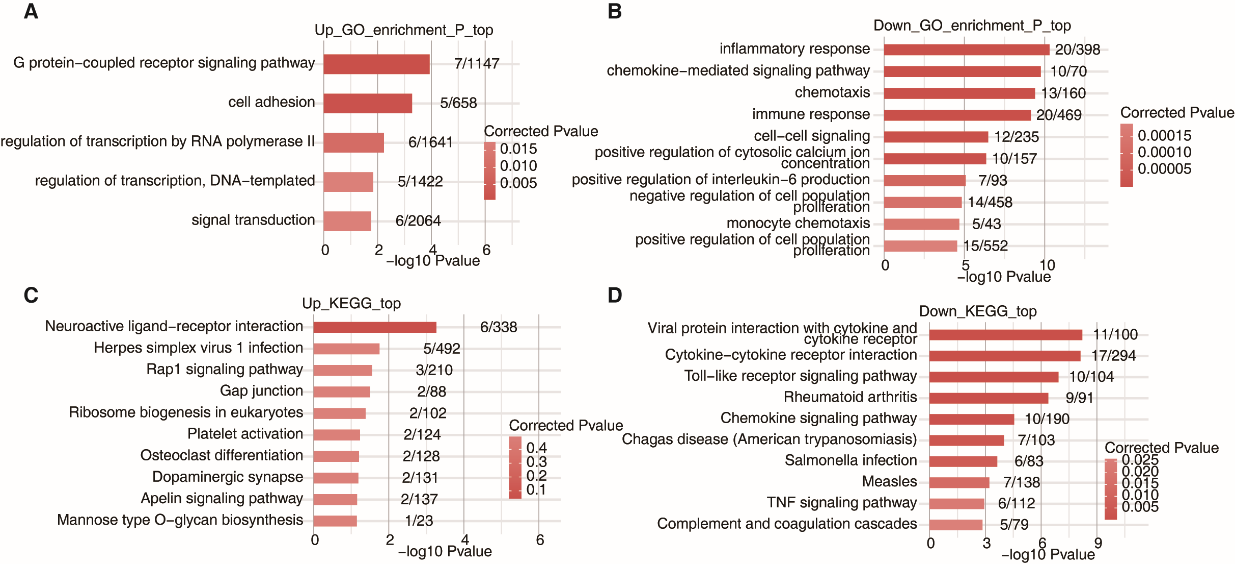
**

**Fig S2. LGALS1 regulates the expression of genes associated with angiogenesis in HRMECs. a** Top 10 most enriched Gene Ontology (GO) terms associated with upregulated genes between the siLGALS1 and control groups. **b** Top 10 most enriched GO terms associated with downregulated genes between the siLGALS1 and control groups. **c** Top 10 most enriched Kyoto Encyclopedia of Genes and Genomes (KEGG) terms associated with upregulated genes between the siLGALS1 and control groups. **d** Top 10 most enriched KEGG terms of downregulated genes between the siLGALS1 and control groups. HRMECs, human retinal microvascular endothelial cells.

**
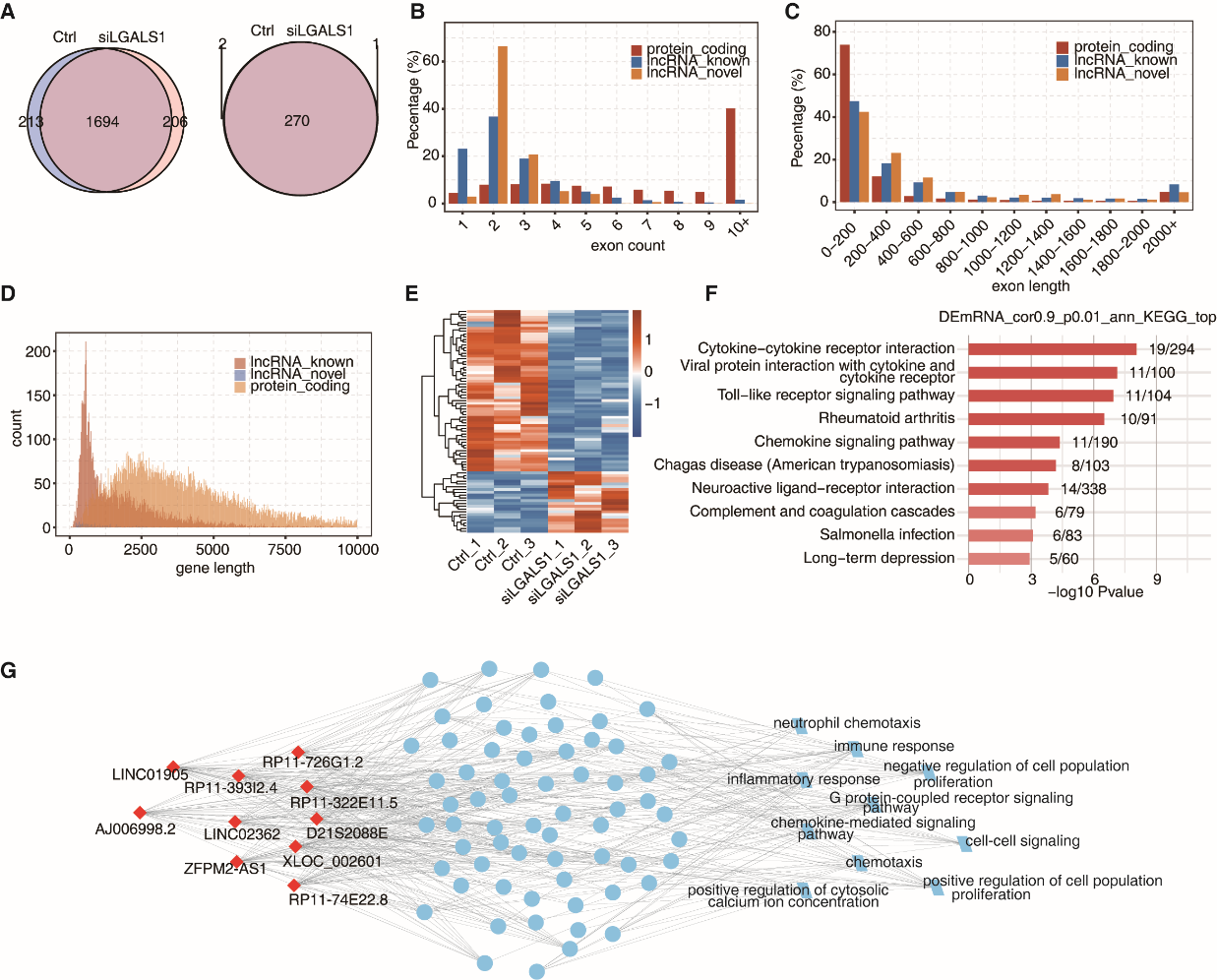
**

**Fig S3. Analysis of differentially expressed lncRNAs in HRMECs between LGALS1-knockdown and control groups. a** Detected known lncRNAs (left panel) and novel lncRNAs (right panel) in the siLGALS1 and control groups. LncRNAs with FPKM ≥ 0.2 in at least one sample were considered differentially expressed. **b** Distribution of exon counts of known lncRNAs, novel lncRNAs, and protein-coding RNAs. **c** Distribution of exon lengths of known lncRNAs, novel lncRNAs, and protein-coding RNAs. **d** Density of the length distribution of known lncRNAs, novel lncRNAs, and protein-coding RNA. The length density distribution was generated using the density function in R software. **e** Expression profile of DE lncRNAs. **f** Top 10 most enriched KEGG pathways associated with DE mRNAs co-expressed with DE lncRNAs in the siLGALS1 and control groups. **g** Co-expression network of DE lncRNAs and DE mRNAs associated with the top 10 GO terms, shown in red in **f**. LncRNAs are on the left, co-expressed mRNAs are in the center, and enriched GO terms associated with mRNAs are on the right. DE, differentially expressed; HRMECs, human retinal microvascular endothelial cells; FPKM, fragments per kilobase of exon per million fragments mapped; KEGG, Kyoto Encyclopedia of Genes and Genomes; GO, Gene Ontology.

**
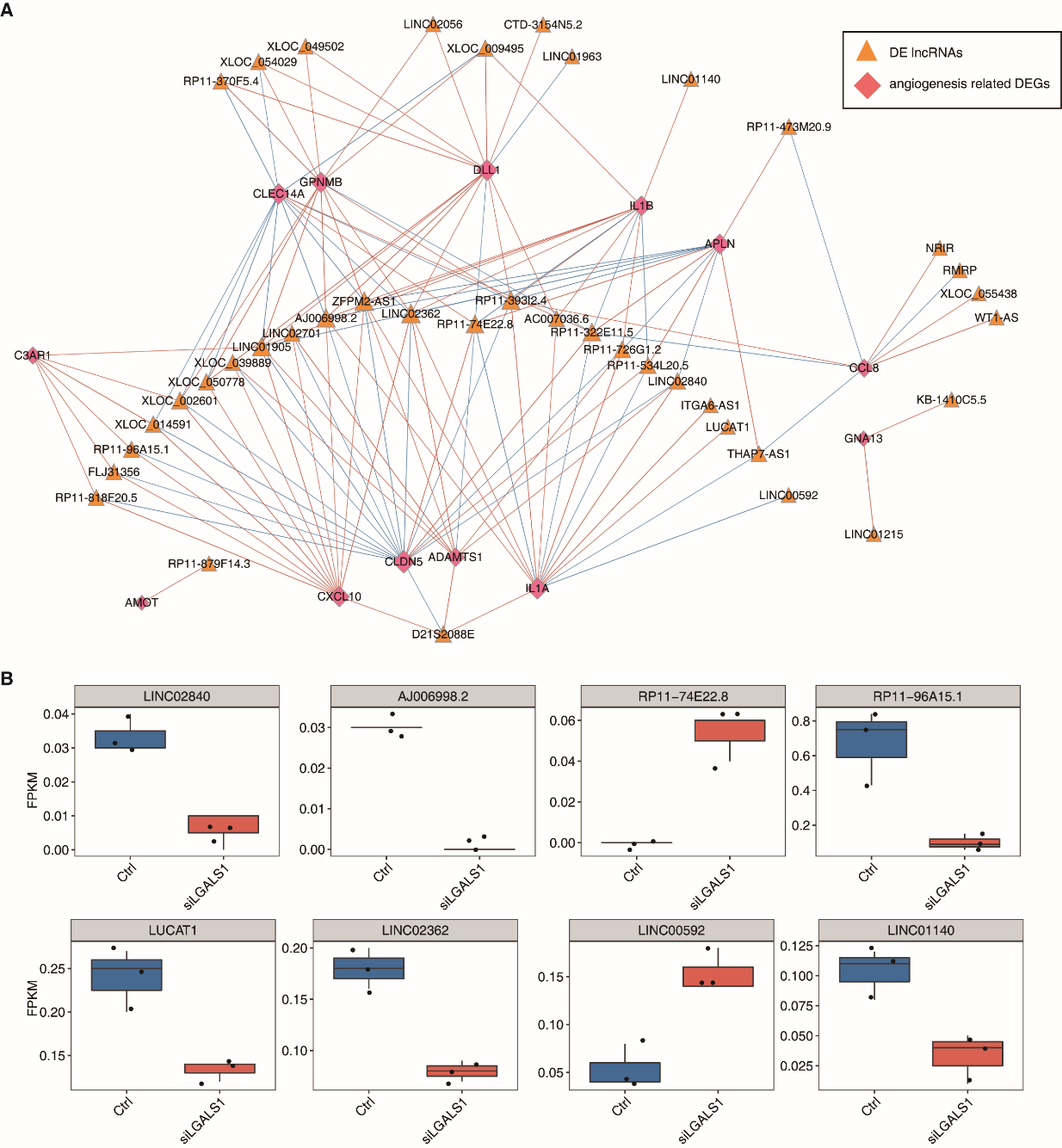
**

**Fig S4. Regulatory network of lncRNA–miRNA–angiogenesis-related genes mediated by LGALS1 deregulation in HRMECs. a** Co-expression network of DE lncRNAs and DE angiogenesis-related genes. Orange triangles indicate lncRNAs, and red rhombuses indicate angiogenesis-related genes. Cutoffs of *p* ≤ 0.01 and PCC ≥ 0.95 were applied to identify co-expression pairs. **b** Box plots showing the expression profiles of the eight DE lncRNAs involved in the regulatory network. HRMECs, human retinal microvascular endothelial cells; DE, differentially expressed; PCC, Pearson’s correlation coefficient.


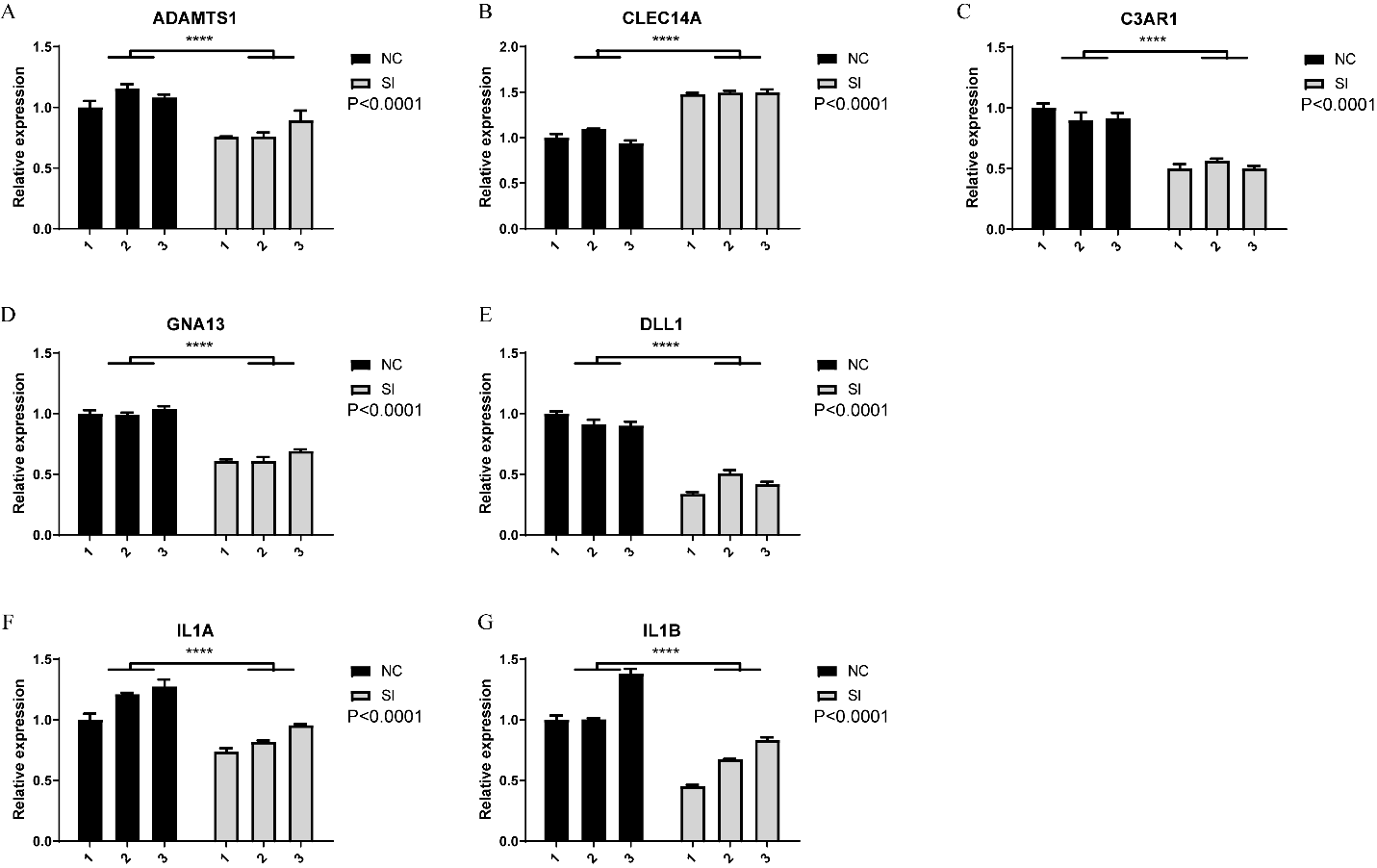


**Fig S5. Validation of the expression of angiogenesis-related genes in HRMECs with or without knockdown LGALS1. a-g** Expression of differentially expressed angiogenesis-related genes involved in the network quantified by qPCR. ****p < 0.0001.

**Table S1. Primer sequences**

| Gene | Primer | Sequence (5'–3') | PCR Product |
| --- | --- | --- | --- |
| Hum GAPDH | Forward | GGTCGGAGTCAACGGATTTG | 218 bp |
|  | Reverse | GGAAGATGGTGATGGGATTTC |  |
| LGALS1 | Forward | CCTGGAAGTGTTGCAGAGGT | 131 bp |

**Table S2. siRNA sequences**

| Number | Gene | Sense (5'–3') sequence | Antisense (5'–3') sequence |
| --- | --- | --- | --- |
| siNC | siNC | UUCUCCGAACGUGUCACGUTT | ACGUGACACGUUCGGAGAATT |
| si-1 | LGALS1-homo-117 | CCAGCAACCUGAAUCUCAATT | UUGAGAUUCAGGUUGCUGGTT |
| si-2 | LGALS1-homo-204 | GCAAAGACAGCAACAACCUTT | AGGUUGUUGCUGUCUUUGCTT |
| si-3 | LGALS1-homo-270 | CCAUCGUGUGCAACAGCAATT | UUGCUGUUGCACACGAUGGTT |
| si-4 | LGALS1-Homo-397 | GCUGCCAGAUGGAUACGAATT | UUCGUAUCCAUCUGGCAGCTT |
| si-5 | LGALS1-Homo-446 | GCCAUCAACUACAUGGCAGTT | CUGCCAUGUAGUUGAUGGCTT |
